# Associations of Cord Blood Lipids with Childhood Adiposity at the Age of Three Years: A Prospective Birth Cohort Study

**DOI:** 10.3390/metabo12060522

**Published:** 2022-06-06

**Authors:** Qi-Qing Ye, Shao-Min Kong, Xin Yin, Chang Gao, Min-Shan Lu, Rema Ramakrishnan, Cheng Guo, Wang Yao, Ji-Yuan Zeng, Ya-Shu Kuang, Jin-Hua Lu, Jian-Rong He, Xiu Qiu

**Affiliations:** 1 Division of Birth Cohort Study, Guangzhou Women and Children’s Medical Center, Guangzhou Medical University, Guangzhou 510623, China; 2018122026@stu.gzhmu.edu.cn (Q.-Q.Y.); shaomin.kong@bigcs.org (S.-M.K.); xinyin@stu.gzhmu.edu.cn (X.Y.); chang.gao@bigcs.org (C.G.); minshan.lu@bigcs.org (M.-S.L.); 2019122029@stu.gzhmu.edu.cn (C.G.); yaow23@mail2.sysu.edu.cn (W.Y.); 2018122016@stu.gzhmu.edu.cn (J.-Y.Z.); yashu.kuang@bigcs.org (Y.-S.K.); jinhua.lu@bigcs.org (J.-H.L.); 2 Paediatrics School, Guangzhou Medical University, Guangzhou 511432, China; 3 National Perinatal Epidemiology Unit, Nuffield Department of Population Health, University of Oxford, Oxford OX3 7LF, UK; rema.ramakrishnan@npeu.ox.ac.uk; 4 Department of Clinical Medicine, Zhongshan School of Medicine, Sun Yat-sen University, Guangzhou 510080, China; 5 Department of Women’s Health, Provincial Key Clinical Specialty of Woman and Child Health, Guangzhou 510623, China; 6 Provincial Clinical Research Center for Child Health, Guangzhou 510623, China

**Keywords:** childhood adiposity, cord blood, lipids, birth weight

## Abstract

We aimed to examine the associations between cord blood lipids and childhood adiposity and to investigate whether these associations vary across birth weight categories (small for gestational age (SGA), appropriate for gestational age (AGA) and large for gestational age (LGA)) in 1306 infants in the Born in Guangzhou Cohort Study, China. Adiposity outcomes at the age of three years included z-scores of weight-for-length/height (WFLZ), body mass index (BMIZ), subscapular (SSTZ) and triceps skinfold thickness (TSTZ), and the sum of skinfold thicknesses (SSFTZ). Cord blood triglycerides (TG) levels were negatively associated with WFLZ and BMIZ, whereas high density lipoprotein (HDL) levels were positively associated with WFLZ, BMIZ, TSTZ and SSFTZ. These associations were attenuated after adjustment for birth weight. Stratified analyses revealed that total cholesterol (TC) and low-density lipoprotein (LDL) levels were positively associated with childhood adiposity indicators among AGA infants but tended to be negatively associated with the adiposity indicators among LGA infants (*p* values for interaction <0.05). Furthermore, TG levels appeared to be positively associated with adiposity indicators among SGA infants but negatively associated with the outcomes among LGA infants (*p* values for interaction <0.05). Cord blood lipids levels might be associated with childhood adiposity, and these associations appear to differ across different birth weight categories. If confirmed in future studies, our findings suggest that individualized management plans might be warranted in preventing obesity.

## 1. Introduction

Childhood obesity is a public health concern, whose prevalence increased from 2% in 1980 to 5% in 2015 globally [1]. In 2015, there were around 108 million obese children around the world, with the major contributors being China and India [2]. Childhood obesity has been associated with multiple short- and long-term negative health outcomes, including advanced sexual and skeletal maturation, increased risk of obesity, cardiovascular diseases, and type 2 diabetes mellitus in adulthood [3]. Therefore, the identification of risk factors for childhood obesity is critical to curb the obesity pandemic and associated non-communicable diseases.

Maternal nutrition and intrauterine metabolic environment play important roles in the development of childhood obesity [4]. The intrauterine environment of hyperglycemia has been considered a crucial factor for fetal overgrowth and childhood obesity. However, clinical studies have found that standard clinical management for gestational diabetes mellitus (GDM) does not reduce the risk of obesity in offspring [5,6]. This suggests that non-glycemic factors during pregnancy might contribute to childhood obesity. Recent studies have proposed that maternal lipid levels (e.g., triglycerides (TGs)) during pregnancy are closely related to fetal growth [7]. Some studies have reported positive associations between maternal lipids and childhood adiposity [8,9], whereas others have reported null findings [10,11,12]. These studies have primarily focused on lipid levels of pregnant women but have not accounted for fetal lipid metabolism, which is likely to be different from that of their mothers.

We have recently reported that infants of different birth weights have distinctive lipids profiles at birth [13]. For example, as compared to neonates born with the appropriate weight for gestational age (AGA), neonates that are small for their gestational age (SGA) were found to have lower levels of total cholesterol (TC), high-density lipoprotein (HDL) and low-density lipoprotein (LDL), and higher levels of TG in cord blood, whereas neonates that are large for their gestational age (LGA) had marginally significant lower levels of TG [13]. However, it is unclear whether these altered lipid profiles in infants with different birth weights play distinct roles in the subsequent growth and risk for childhood obesity.

In this prospective cohort study in China, we aimed to investigate the associations between cord blood lipid levels and child adiposity indicators at the age of 3 and further to assess if these associations differed by birth weight categories (i.e., SGA, AGA and LGA).

## 2. Results

Baseline characteristics of 1306 mother–infant pairs are shown in Table 1. Most (65%) women had normal body mass index (BMI) before pregnancy, and only 11% of them were overweight or obese. Approximately 12.5% of women were diagnosed with GDM, and 3.7% had pregnancy-induced hypertension. A total of 4.4% of the children were born preterm. The prevalence of SGA and LGA was 6.5% and 6.6%, respectively.

### 2.1. Associations of Cord Blood Lipids and Childhood Adiposity Indicators

Table 2 presents the associations between cord blood lipids and childhood adiposity indicators in the total population. After adjustment for maternal age, parity, educational level, pre-pregnancy BMI, GDM and pregnancy-induced hypertension, it was found that cord blood TG levels were negatively associated with weight-for-length/height z-score (WFLZ) (β, −0.08; 95% CI, −0.13 to −0.02) and body mass index z-score (BMIZ) (β, −0.07; 95% CI, −0.13 to −0.02). To investigate whether the associations between lipids measures and child adiposity were independent of the child’s birth weight, we further included birth weight in the model for adjustment (model 2). Notably, these associations for TG were attenuated after adjustment for birth weight (model 2). Cord blood HDL levels were positively associated with WFLZ (β, 0.06; 95% CI, 0.01 to 0.12), BMIZ (β, 0.06; 95% CI, 0.00 to 0.11), triceps skinfold thickness z-score (TSTZ) (β, 0.10; 95% CI, 0.02 to 0.18) and the sum of the skinfold thicknesses’ z-score (SSFTZ) (β, 0.15; 95% CI, 0.01 to 0.29). However, only the association with TSTZ was retained after the adjustment for birth weight (model 2).

### 2.2. Stratified Associations by Birth Weight Categories

Table 3 displays the associations of cord blood lipids and child adiposity indicators by birth weight subgroups. Cord blood TC and LDL levels were positively associated with WFLZ, BMIZ and subscapular skinfold thickness z-score (SSTZ) among AGA infants. Associations for these lipid measures and adiposity indicators appear to be negative among LGA infants, with the upper limits of the 95% CI of the regression coefficients slightly above zero. For example, the regression coefficients (95% CI) of LDL among AGA and LGA infants were 0.06 (0.00 to 0.13) and −0.16 (−0.34 to 0.02) for WFLZ, 0.06 (0.00 to 0.13) and −0.18 (−0.36 to 0.01) for BMIZ, 0.13 (0.01 to 0.25) and −0.48 (−0.79 to −0.17) for SSTZ and 0.04 (−0.06, 0.13) and −0.30 (−0.62, 0.03) for TSTZ. Interaction analysis showed statistically significant differences for these associations between AGA and LGA infants (*p* values for interaction, <0.05).

Interaction analysis also showed that the associations of TG with WFLZ and BMIZ were different between LGA and SGA infants (*p* values for interaction, <0.05). Cord blood TG levels were negatively associated with WFLZ and BMIZ among LGA infants. On the other hand, TG levels tended to be positively associated with these outcomes among SGA infants, but the confidence intervals of the regression coefficients are wide and cover zero.

### 2.3. Other Stratified Analysis and Sensitivity Analyses

Overall, the associations between cord blood lipids and childhood adiposity indicators were similar between women with and without GDM (Table S1) and between women who were underweight, normal-weight and overweight/obese before pregnancy (Tables S2 and S3). In sensitivity analyses, the results were similar to the primary analysis when children were born preterm (*n* = 58) (Table S4) or women with pregnancy-induced hypertension (*n* = 48) (Table S5) were excluded.

## 3. Discussion

### 3.1. Main Findings

This is the first study that examined the association of various cord blood lipids and childhood adiposity measured at the age of three. At first, only TG and HDL-C were found to be associated with several adiposity indicators, and such associations were mostly no longer significant after adjusting for the birth weight of the children. After stratification by birth weight categories, the associations between cord blood lipids and childhood adiposity differed across birth weight groups (SGA, AGA and LGA). Specifically, TC and LDL levels were positively associated with childhood adiposity indicators among AGA infants but negatively associated with the adiposity indicators among LGA infants with marginal statistical significance. Furthermore, TG levels were negatively associated with adiposity indicators among LGA infants but appeared to be positively associated with the outcomes among SGA infants with marginal statistical significance.

### 3.2. Interpretation of the Results

The relationship between cord blood lipid profile and childhood obesity or adiposity has been rarely studied, and available evidence has focused specifically on the fatty acid composition of cord blood rather than the absolute content of lipids. It has been shown by Donahue et al. [14] that both the relative proportion of n-3 polyunsaturated fatty acids (PUFA) and the n-6/n-3 PUFA ratio in cord blood plasma are associated with skinfold thickness of children as measured at the age of three, which might be related to the adipogenic effect of different PUFA, as previously demonstrated in animal model [15]. However, other studies found no correlation between the red blood cell PUFA profile and skinfolds or fat mass of children at one year of age [16] or between plasma phospholipids PUFA composition and overweight or obesity measured at the age of 5 [17], respectively.

Unlike the aforementioned studies, our study used the absolute concentration of various lipid parameters rather than the relative proportion of fatty acids against the total fatty acid pool, which might be more indicative of the metabolic environment that the neonate exposed to in utero at the very last stage of gestation. We found that cord blood TG was negatively associated with WFLZ and BMIZ and that HDL-C was positively associated with WFLZ, TSTZ and SSFTZ without adjustment for birth weight. This is contradictory to a previous report in which the authors found a negative association between cord blood HDL-C and infant weight at six months of age [11]. Theoretically, large molecules such as HDL-C cannot cross the placenta directly, but their presence and concentration could interfere with the metabolism of other lipids [18]. The potential interaction between various lipids might be critical for the neonatal metabolism, which may have lasting impact on the metabolic health of children, highlighting a potential area for further research. Our results regarding the association between cord blood TG and childhood adiposity are broadly consistent with previous reports, where TG was found to be negatively correlated with newborn adiposities [13,19] and childhood BMI (in both cross-sectional evaluation and longitudinal analysis) [20]. When the models were adjusted for birth, we found that only the association between HDL-C and TSTZ remained significant. This suggests that associations of cord blood lipids with childhood adiposity were dependent of the infant’s birth weight. However, the exact role of birth weight (mediator vs. confounder) is difficult to assess in our current data because both cord blood lipids and birth weight were measured at the same time point (i.e., at birth).

It has been reported that cord blood lipid profile is associated with birth weight or sufficiency of intrauterine growth [21,22], implying that a stratified analysis based on birth weight categories is warranted to better dissect the complex relation between cord blood lipids, birth weight group and childhood adiposities. In the present study, we found that the associations between cord blood TG levels and childhood adiposity were different between LGA and SGA infants: there were negative associations in LGA infants but trends toward positive associations in SGA infants. We speculate that these results can be partially explained by the well-established interaction between intrauterine nutritional and metabolic status and infant birth weight. SGA newborns might have intrauterine growth restriction (IUGR) [23], which can limit β-cell replication and result in lower fetal insulin secretion and poor fat synthesis and deposition [24]. It has also been shown that IUGR newborns have a lower level of fetus’ lipoprotein lipase (LPL) [25], which is known to serve as the key enzyme in promoting TG metabolism, existing mainly in adipose tissue [26]. Therefore, higher circulating TG levels in SGA newborns might reflect lower LPL activity and less fetal fat accumulation. Due to this intrauterine metabolic disorder, SGA fetuses are more prone to elevated body fat percentage and ectopic fat deposition during postnatal catch-up development [13], increasing the risk of obesity and the future development of cardiometabolic diseases [27,28]. In contrast, we found that LGA newborns had lower TG levels at birth, which reflects well-developed adipose tissue in the fetus and overnutrition in uterine environment [29]. Animal studies showed that overnutrition could cause insulin and leptin resistance in the hypothalamus via various pathways (e.g., oxidative stress, neurodegeneration and the activation of hypothalamic IKK beta/NF-kappa B paths) [30,31]. These changes in the hypothalamus may lead to incompetence in terms of appetite inhibition, increased food intake and subsequent adiposity after birth. Therefore, lower levels of cord blood TG, representing fetal overgrowth among LGA infants, are associated with higher childhood adiposity among LGA infants.

Interestingly, we observed positive associations between cord blood TC and LDL-C and adiposity indicators of those born AGA but negative associations (with marginal statistical significance) were found between these factors in children born LGA. Cholestenone, a cholesterol metabolic product in cord blood has previously been associated with rapid weight gain in infants within the first year of life, defined as an increase in weight z-score for more than 0.67 from birth to one year, and this association was independent of birth weight. However, animal models have demonstrated the anti-obesogenic effect of cholestenone supplementation, which prevented the accumulation of fat mass [32], possibly via inflammation suppression [33]. Given that we have previously demonstrated a linear relationship between cord blood TC and LDL-C and birth weight [13], it remains to be further investigated whether there is a tipping point between the level of cholesterol (and/or its metabolites) and childhood adiposity and/or obesity and whether such a careful balance would explain the results seen in our study.

### 3.3. Implications

Recent studies have proposed that lipids during pregnancy are closely related to increased fetal growth [7]. Our observation that cord blood lipids levels are associated with childhood adiposity indicators further supports that the intrauterine lipid environment might play a role in the development of childhood obesity. In addition, we found that the associations between cord blood lipids levels and childhood adiposity were different among children born SGA, AGA and LGA. Although the mechanisms remain unclear, our findings support the notion that infant with different birth weights might have different in utero metabolic profiles and different weight management strategies should be developed for these infants to optimize child growth and reduce the risk of childhood obesity.

### 3.4. Strengths and Limitations

Our study has a few strengths. The prospective cohort design of our study can reduce the risk of recall bias on data collection. A relatively large sample size ensures sufficient statistical power to explore the relationship between the cord blood lipid profile and the childhood adiposity. We were also able to adjust for several potential confounding factors in the analysis, using data from a well-characterized birth cohort.

However, the limitations of our study should also be acknowledged. First, our study population was mainly recruited from a medical center in Guangzhou, and thus, our findings might not be extrapolated to the other populations in China. Second, the anthropometric data of the most recent measurements in routine child care were collected through parental telephone interviews or electronic health record system for about 40% of children. This might have caused potential recall bias or heterogeneity in the growth measurements. Third, a self-reported questionnaire was used to collect data on maternal pre-pregnancy weight and height, which might have led to recall bias. Thirdly, regarding cord blood lipids measurements, we only included the absolute content of lipids. This study could be more complete if both the fatty acid composition and the absolute content of lipids were to be included, considering their diverse biological functions. Fourth, there are a small number of childhood obesity cases in our sample; thus, we were unable to construct a prediction model for childhood obesity, which is clinically relevant. Lastly, data on skinfold thickness were only available in less than half of the children, which might have resulted in selection bias in the analysis for this outcome.

## 4. Materials and Methods

### 4.1. Study Population

This study was part of the Born in Guangzhou Cohort Study (BIGCS), which is a prospective study launched in 2012, based in the Guangzhou Women’s and Children’s Medical Center (GWCMC). The study design and cohort profile of BIGCS have been described previously [34]. Pregnant women were invited for participation in BIGCS at their first antenatal care in GWCMC if they were at less than 20 weeks of gestation, intended to give birth in GWCMC and planned to reside in Guangzhou for at least 3 years after delivery. After enrollment, the pregnant women were followed up in second and third trimesters, and the mother–child pairs were followed up at multiple time points postnatally. Epidemiological and clinical data were gathered via self-administered questionnaires, clinical assessments and/or medical records. The protocol of BIGCS was approved by the GWCMC Ethics Committee, and all participants provided written consent.

Inclusion criteria for the current study were (1) available cord blood samples, (2) available fasting blood samples in mid-pregnancy and data on oral glucose tolerance test (OGTT), (3) no severe disease before pregnancy (e.g., type 1 or type 2 diabetes, pregestational hypertension and kidney diseases) and (4) available data on maternal characteristics. A total of 1744 mother–child pairs were randomly selected from all eligible women. This sample has been used in our previous study [35]. We do not have an a priori sample size calculation. We further excluded infants with severe birth defects (*n* = 8) and without data on child growth at age of 3 years (*n* = 403) and covariates (*n* = 27). Finally, 1306 mother–child pairs were included in the current analysis.

### 4.2. Child Adiposity Indicators

Adiposity outcomes included WFLZ, BMIZ, SSTZ, TSTZ and SSFT. Z-scores of WFLZ, BMIZ, SSTZ and TSTZ were calculated based on the Chinese child growth standards [36]. The Z-score of SSFT was calculated using internal mean and standard deviation.

Trained research assistants obtained anthropometric measurements at the 3-year follow-up visit (30–42 months). Before measurements, the child was asked to take off their shoes and only keep single-layer clothes. The weight and length of children under 36 months were measured using a Shekel Healthweight™ Scale (Shekel Scales Ltd., Lower Galilee, Israel) in supine position, whereas the weight and height of children at 36 months or older were measured using a Seca 287 digital scale in (Seca GmbH & Co. KG, Hamburg, Germany) standing position. Weight was recorded in kilograms with two decimals, and length/height was recorded in centimeters with one decimal place. After measuring, the weight of clothing (200 g) was subtracted from the weight of each child. Subscapular and triceps skinfold thicknesses were measured using Harpenden skinfold calipers (Baty International, West Sussex, UK). The sum of the skinfold is the summation of triceps and subscapular skinfold thickness. If the child was unable to attend an in-person follow-up visit in BIGCS, their anthropometric data (i.e., height/length, weight) of the most recent measurement in routine child care were collected through parental telephone interviews or cross-referenced from the electronic health record system (about 40% in the current sample). The main reason for this non-attendance was reduced motivation for the follow-up at the cohort clinic because the child had recently had visits at other child care clinics. However, data on skinfold thickness were only available among 576 children.

### 4.3. Cord Blood Lipids Measurements

Venous umbilical cord blood samples were collected at birth using anticoagulant ethylenediaminetetraacetic acid (EDTA) tubes. Blood samples were centrifuged within 24 h after being collected to separate plasma, which were then stored at −80 °C until analysis. Blood lipid profile was measured in cord blood plasma samples including TC, TG, HDL and LDL. All assays had low intra-day and inter-day coefficients of variation (<2%). Data on cord blood lipids have been used in our previous studies [35]. 

### 4.4. Covariates

Data on covariates were obtained from questionnaires completed by pregnant women at recruitment, including age (continuous), educational level (middle school or below, vocational or technical college, undergraduate or postgraduate), pre-pregnancy weight (continuous), height (continuous) and parity (primipara/multipara). Information regarding maternal pregnancy complications (i.e., GDM and pregnancy-induced hypertension), birth weight and sex of the child were sourced from medical records. SGA, AGA and LGA were defined as birth weight of below 10th, 10th to 90th, and above 90th gestational age- and sex-specific percentiles based on the INTERGROWTH-21st standards, respectively [37]. The pre-pregnancy weight and height were utilized to calculate BMI before pregnancy (BMI = weight (kg)/height (m)^2^). The diagnosis of GDM was based on the by the International Association for Diabetes and Pregnancy Research guideline [38,39]. Pregnancy-induced hypertension was ascertained using the International Classification of Diseases, Tenth Revision (ICD-10) codes O13–O16.

### 4.5. Statistical Analysis

We log-transformed the lipid data to address their skew distributions and then converted them to z-scores based on the mean and standard deviation in the analytic sample to improve the comparability of results between metabolic factors, as they have different units and scales. Figure S1 shows the distribution of lipid measures before and after transformation. 

A multiple linear regression model was used to examine the associations between the level of each cord blood metabolic factor (continuous) and childhood adiposity indicators (including BMIZ, WFLZ, SSTZ and TSTZ). Regression coefficients (β) and 95% confidence intervals (CIs) were obtained from two models: model 1 was adjusted for maternal age, parity, education level, pre-pregnancy BMI, GDM, pregnancy-induced hypertension and child’s sex; model 2 was additionally adjusted for birth weight to investigate whether the associations between metabolic factors and child adiposity were independent of the child’s birth weight.

To further explore whether the associations between cord blood lipids and child adiposity differed among infants with different birth weights, we stratified the association analysis by birth weight categories (SGA, AGA and LGA). The interaction between lipid measures and birth weight categories on child adiposity was tested by including a product term in the model. We also performed exploratory stratified analyses by maternal GDM status (yes or no) and pre-pregnancy BMI (underweight, normal and overweight/obese) to investigate whether the association between lipid measures and child adiposity varies by maternal metabolic status.

In addition, to assess the impacts of preterm birth and pregnancy-induced hypertension on the results, we carried out two sensitivity analyses by excluding preterm infants and hypertensive women, respectively. All analyses were performed using SPSS 21.0, and the statistical significance was set at two-tailed *p* < 0.05. All analyses were complete case analyses without imputing missing values. 

## 5. Conclusions

Our study shows that cord blood lipids were associated with childhood adiposity, and these associations might be different among infants of different birth weights. Given the limitations of our study, future studies are needed to confirm our findings, which might have implications in tailored monitoring or intervention strategies for optimal child growth and obesity prevention.

## Figures and Tables

**Table 1:** Characteristics of mothers and children.

Variables	All *N* = 1306	Boys *N* = 699	Girls *N* = 607
Maternal characteristics			
Age mean (SD), years	29.8 (3.4)	29.9 (3.5)	29.7 (3.2)
Educational level (%)			
Middle school or below	77 (5.9)	38 (5.4)	39 (6.4)
College	252 (19.3)	141 (20.2)	111 (18.3)
Undergraduate	773 (59.2)	417 (59.7)	356 (58.6)
Postgraduate	204 (15.6)	103 (14.7)	101 (16.6)
Pre-pregnancy BMI			
Mean (SD), kg/m^2^	20.5 (2.7)	20.5 (2.7)	20.6 (2.7)
Underweight (%)	310 (23.7)	172 (24.6)	138 (22.7)
Normal weight (%)	853 (65.3)	450 (64.4)	403 (66.4)
Overweight/obesity (%)	143 (10.9)	77 (11.0)	66 (10.9)
Nulliparous (%)	1003 (76.8)	532 (76.1)	471 (77.6)
GDM (%)	163 (12.5)	81 (11.6)	82 (13.5)
Pregnancy-induced hypertension (%)	48 (3.7)	25 (3.6)	23 (3.8)
Child’s characteristics			
Gestational age			
Median (25th–75th), weeks	39.0 (38.0, 40.0)	39.0 (38.0, 40.0)	39.0 (38.0, 40.0)
Preterm (%)	58 (4.4)	38 (5.4)	20 (3.3)
Term (%)	1248 (95.6)	661 (94.6)	587 (96.7)
Birth weight			
SGA (%)	85 (6.5%)	40 (5.7%)	45 (7.4%)
AGA (%)	1135 (86.9%)	617 (88.3%)	518 (85.3%)
LGA (%)	86 (6.6%)	42 (6.0%)	44 (7.2%)
Child growth measurements			
Age at measurement			
Mean (SD), months	34.7 (3.2)	34.7 (3.1)	34.7 (3.3)
Length mean (SD), cm	94.9 (7.5)	95.6 (7.0)	94.1 (8.0)
Weight mean (SD), kg	13.9 (2.8)	14.1 (2.1)	13.7 (3.3)
BMI mean (SD), kg/m^2^	15.2 (1.23)	15.3 (1.21)	15,1 (1.23)
SST mean (SD), mm	6.8 (1.9)	6.7 (2.0)	7.0 (1.8)
TST mean (SD), mm	10.1 (2.3)	10.1 (2.3)	10.1 (2.2)
Lipids level in cord blood, median (25th–75th)			
Total cholesterol, mmol/L	1.68 (1.44–1.96)	1.61 (1.36–1.92)	1.73 (1.51–2.00)
Triglycerides, mmol/L	0.33 (0.27–0.41)	0.33 (0.27–0.41)	0.33 (0.27–0.41)
HDL, mmol/L	0.87 (0.72–1.05)	0.83 (0.70–1.02)	0.92 (0.74–1.10)
LDL, mmol/L	0.58 (0.45–0.73)	0.56 (0.42–0.71)	0.60 (0.47–0.75)

SD = standard deviation. BMI = body mass index; GDM = gestational diabetes mellitus; TST = triceps skinfold thickness z-score; SST = subscapular skinfold thickness; HDL = high-density lipoprotein; LDL = low-density lipoprotein; SGA = small for gestational age; AGA = appropriate for gestational age; LGA = large for gestational age.

**Table 2:** Associations of cord blood lipids with childhood adiposity indicators in the whole study population.

Cord Blood Metabolic Factors	Model 1		Model 2	
	Beta (95% CI)	*p*	Beta (95% CI)	*p*
Total cholesterol				
Weight-for-length z-score	0.03 (−0.02, 0.09)	0.23	0.02 (−0.04, 0.07)	0.56
BMI-for-age z-score	0.03 (−0.02, 0.09)	0.27	0.02 (−0.04, 0.07)	0.59
TST-for-age z-score	0.06 (−0.02, 0.14)	0.15	0.06 (−0.03, 0.14)	0.18
SST-for-age z-score	0.09 (−0.02, 0.19)	0.12	0.07 (−0.03, 0.18)	0.18
SSFT-for-age z-score	0.10 (−0.04, 0.24)	0.16	0.09 (−0.05, 0.23)	0.21
Triglycerides				
Weight-for-length z-score	−0.08 (−0.13, −0.02)	<0.01	0.00 (−0.05, 0.06)	0.94
BMI-for-age z-score	−0.07 (−0.13, −0.02)	0.01	0.00 (−0.06, 0.06)	0.96
TST-for-age z-score	−0.04 (−0.12, 0.04)	0.29	−0.03 (−0.11, 0.05)	0.46
SST-for-age z-score	−0.08 (−0.18, 0.03)	0.15	−0.04 (−0.15, 0.07)	0.49
SSFT-for-age z-score	−0.13 (−0.27, 0.00)	0.06	−0.10 (−0.24, 0.04)	0.17
HDL				
Weight-for-length z-score	0.06 (0.01, 0.12)	0.03	0.03 (−0.03, 0.08)	0.30
BMI-for-age z-score	0.06 (0.00, 0.11)	0.05	0.03 (−0.03, 0.08)	0.32
TST-for-age z-score	0.10 (0.02, 0.18)	0.01	0.10 (0.02, 0.18)	0.02
SST-for-age z-score	0.08 (−0.03, 0.19)	0.14	0.06 (−0.04, 0.17)	0.23
SSFT-for-age z-score	0.15 (0.01, 0.29)	0.04	0.13 (−0.01, 0.27)	0.06
LDL				
Weight-for-length z-score	0.03 (−0.03, 0.08)	0.33	0.02 (−0.04, 0.07)	0.54
BMI-for-age z-score	0.03 (−0.03, 0.08)	0.35	0.02 (−0.04, 0.07)	0.55
TST-for-age z-score	0.00 (−0.08, 0.08)	0.95	−0.01 (−0.09, 0.08)	0.90
SST-for-age z-score	0.08 (−0.03, 0.19)	0.14	0.07 (−0.04, 0.18)	0.20
SSFT-for-age z-score	−0.02 (−0.16, 0.12)	0.73	−0.03 (−0.17, 0.11)	0.64

BMI = body mass index; TST = triceps skinfold thickness z-score; SST = subscapular skinfold thickness; SSFT = sum of skinfold thicknesses; HDL = high-density lipoprotein; LDL = low-density lipoprotein. Model 1 adjusted for maternal age, parity, educational level, pre-pregnancy BMI, GDM, pregnancy-induced hypertension, child sex; Model 2 adjusted for maternal age, parity, educational level, pre-pregnancy BMI, GDM, pregnancy-induced hypertension, child sex, and birth weight.

**Table 3:** Stratified associations between cord blood lipids and childhood adiposity indicators by birth weight categories.

Cord Blood Lipids	Regression Coefficients (95% Confidence Interval)			*p*_interaction_		
	SGA (*N* = 85)	AGA (*N* = 1135)	LGA (*N* = 86)	SGA vs. AGA	LGA vs. AGA	SGA vs. LGA
Total cholesterol						
Weight-for-length z-score	0.07 (−0.12, 0.25)	0.06 (0.01, 0.12)	−0.15 (−0.35, 0.04)	0.55	0.01	0.16
BMI-for-age z-score	0.08 (−0.11, 0.26)	0.06 (0.00, 0.12)	−0.17 (−0.36, 0.03)	0.61	<0.01	0.12
TST-for-age z-score	−0.01 (−0.34, 0.32)	0.09 (−0.01, 0.18)	−0.08 (−0.47, 0.32)	0.15	0.24	0.86
SST-for-age z-score	0.02 (−0.41, 0.45)	0.13 (0.01, 0.25)	−0.39 (−0.78, 0.00)	0.17	0.04	0.55
SSFT-for-age z-score	−0.02 (−0.36, 0.32)	0.11 (−0.02, 0.23)	0.09 (−1.78, 1.96)	0.75	0.18	0.16
Triglycerides						
Weight-for-length z-score	0.12 (−0.06, 0.31)	−0.04 (−0.10, 0.02)	−0.25 (−0.46, −0.05)	0.09	0.08	0.01
BMI-for-age z-score	0.12 (−0.07, 0.30)	−0.04 (−0.10, 0.03)	−0.25 (−0.46, −0.04)	0.11	0.09	0.02
TST-for-age z-score	0.01 (−0.28, 0.30)	−0.03 (−0.12, 0.06)	−0.31 (−0.61, 0.00)	0.75	0.23	0.28
SST-for-age z-score	0.06 (−0.34, 0.46)	−0.09 (−0.21, 0.03)	−0.08 (−0.43, 0.26)	0.18	0.66	0.46
SSFT-for-age z-score	0.05 (−0.35, 0.24)	−0.04 (−0.16, 0.09)	−1.01 (−2.43, 0.41)	0.78	0.47	0.42
HDL						
Weight-for-length z-score	0.09 (−0.08, 0.27)	0.07 (0.01, 0.13)	−0.11 (−0.34, 0.11)	0.92	0.07	0.19
BMI-for-age z-score	0.09 (−0.09, 0.26)	0.06 (0.00, 0.12)	−0.12 (−0.35, 0.11)	0.89	0.07	0.19
TST-for-age z-score	−0.04 (−0.31, 0.22)	0.11 (0.02, 0.20)	0.22 (−0.17, 0.61)	0.23	0.76	0.29
SST-for-age z-score	−0.04 (−0.39, 0.30)	0.11 (−0.01, 0.23)	−0.18 (−0.64, 0.29)	0.17	0.13	0.75
SSFT-for-age z-score	−0.04 (−0.31, 0.23)	0.09 (−0.04, 0.22)	1.15 (−0.79, 3.10)	0.29	0.47	0.19
LDL						
Weight-for-length z-score	0.02 (−0.18, 0.21)	0.06 (0.00, 0.13)	−0.16 (−0.34, 0.02)	0.23	<0.01	0.28
BMI-for-age z-score	0.04 (−0.16, 0.23)	0.06 (0.00, 0.13)	−0.18 (−0.36, 0.01)	0.31	<0.01	0.19
TST-for-age z-score	0.07 (−0.27, 0.41)	0.04 (−0.06, 0.13)	−0.30 (−0.62, 0.03)	0.27	0.04	0.48
SST-for-age z-score	0.14 (−0.29, 0.58)	0.13 (0.01, 0.25)	−0.48 (−0.79, −0.17)	0.33	0.02	0.26
SSFT-for-age z-score	0.07 (−0.27, 0.42)	0.11 (−0.02, 0.23)	−1.43 (−2.86, −0.01)	0.54	0.10	0.30

BMI = body mass index; TST = triceps skinfold thickness z-score; SST = subscapular skinfold thickness; SSFT = sum of skinfold thicknesses; HDL = high-density lipoprotein; LDL = low-density lipoprotein; SGA = small for gestational age; AGA = appropriate for gestational age; LGA = large for gestational age. All models were adjusted for maternal age, parity, educational level, pre-pregnancy BMI, GDM, pregnancy-induced hypertension and child sex.

## Data Availability

All data are shown in the paper. No additional data are available. Original data are not shared because of national and regional data regulation policies.

## Supplementary Materials

The following supporting information can be downloaded at: https://www.mdpi.com/article/10.3390/metabo12060522/s1, Table S1: Stratified associations between cord blood metabolic factors and childhood adiposity indicators by maternal GDM status; Table S2: Stratified associations between cord blood metabolic factors and childhood adiposity indicators by BMI z-score; Table S3: Stratified associations between cord blood metabolic factors and childhood adiposity indicators by BMI z-score, additionally adjusted for birth weight z-score; Table S4: Associations between cord blood metabolic factors and childhood adiposity indicators after excluding premature infants; Table S5: Associations between cord blood metabolic factors and childhood adiposity indicators after excluding women with pregnancy-induced hypertension; Figure S1: Distribution of lipid measures before and after transformation.
