# Supplementary material for: Associations of Cord Blood Lipids with Childhood Adiposity at the Age of Three Years: A Prospective Birth Cohort Study
Source: Metabolites. 2022 Jun 6;12(6):522. doi: 10.3390/metabo12060522 (PMC9231066; doi:10.3390/metabo12060522)
Supplement: Supplementary file 1 [file metabolites-12-00522-s001.zip › metabolites-1738209-supplementary.pdf]

Table S1. Stratified associations between cord blood metabolic factors and childhood adiposity indicators by maternal GDM status.

Cord blood lipids	Model 1			Model 2		
	GDM	non-GDM	P interaction	GDM	non-GDM	P interaction
**Total cholesterol**						
Weight-for-length z-score	-0.02 (-0.17, 0.14)	0.05 (-0.01, 0.11)	0.34	-0.02 (-0.17, 0.13)	0.03 (-0.03, 0.08)	0.46
BMI-for-age z-score	-0.02 (-0.17, 0.13)	0.04 (-0.02, 0.10)	0.37	-0.02 (-0.17, 0.13)	0.03 (-0.03, 0.08)	0.48
TST-for-age z-score	-0.04 (-0.30, 0.21)	0.07 (-0.02, 0.15)	0.46	-0.04 (-0.30, 0.21)	0.06 (-0.03, 0.15)	0.51
SST-for-age z-score	-0.01 (-0.36, 0.33)	0.08 (-0.03, 0.19)	0.58	0.00 (-0.35, 0.35)	0.06 (-0.05, 0.18)	0.71
SSFT-for-age z-score	-0.05 (-0.34, 0.24)	0.11 (-0.05, 0.26)	0.43	-0.05 (-0.34, 0.24)	0.09 (-0.07, 0.25)	0.51
**Triglycerides**						
Weight-for-length z-score	-0.05 (-0.21, 0.11)	-0.08 (-0.14, -0.02)	0.60	-0.03 (-0.19, 0.13)	0.01 (-0.05, 0.07)	0.91
BMI-for-age z-score	-0.06 (-0.22, 0.10)	-0.07 (-0.13, -0.01)	0.76	-0.04 (-0.20, 0.12)	0.01 (-0.05, 0.07)	0.80
TST-for-age z-score	0.12 (-0.13, 0.36)	-0.07 (-0.15, 0.02)	0.13	0.12 (-0.13, 0.37)	-0.06 (-0.14, 0.03)	0.15
SST-for-age z-score	0.16 (-0.17, 0.49)	-0.12 (-0.23, -0.01)	0.06	0.17 (-0.16, 0.51)	-0.08 (-0.19, 0.04)	0.08
SSFT-for-age z-score	0.15(-0.13,0.42)	-0.18 (-0.32, -0.03)	0.06	0.15 (-0.13, 0.43)	-0.14 (-0.29, 0.01)	0.08
**HDL**						
Weight-for-length z-score	-0.02 (-0.18, 0.15)	0.08 (0.02, 0.14)	0.19	-0.01(-0.17, 0.15)	0.04 (-0.02, 0.10)	0.43
BMI-for-age z-score	-0.02 (-0.19, 0.14)	0.07 (0.01, 0.13)	0.21	-0.02 (-0.18, 0.15)	0.04 (-0.02, 0.10)	0.43
TST-for-age z-score	-0.01 (-0.28, 0.26)	0.11 (0.02, 0.19)	0.42	-0.01 (-0.28, 0.27)	0.10 (0.02, 0.19)	0.46
SST-for-age z-score	-0.04 (-0.42, 0.33)	0.08 (-0.03, 0.19)	0.40	-0.03 (-0.41, 0.36)	0.06 (-0.05, 0.17)	0.53
SSFT-for-age z-score	-0.04 (-0.35, 0.27)	0.16 (0.14, 0.31)	0.24	-0.05 (-0.37, 0.28)	0.14 (-0.01, 0.29)	0.31
**LDL**						
Weight-for-length z-score	-0.02 (-0.18, 0.13)	0.04 (-0.02, 0.10)	0.42	-0.03 (-0.18, 0.13)	0.03 (-0.03, 0.08)	0.44
BMI-for-age z-score	-0.02 (-0.18, 0.14)	0.04 (-0.02, 0.10)	0.45	-0.02 (-0.18, 0.13)	0.03 (-0.03, 0.09)	0.47
TST-for-age z-score	-0.09 (-0.33, 0.16)	0.00 (-0.09, 0.09)	0.60	-0.09 (-0.33, 0.16)	0.00 (-0.09, 0.09)	0.65

SST-for-age z-score	-0.06 (-0.39, 0.28)	0.08 (-0.03, 0.19)	0.56	-0.05 (-0.39, 0.28)	0.07 (-0.05, 0.18)	0.66
SSFT-for-age z-score	-0.09 (-0.36, 0.19)	-0.04 (-0.19, 0.12)	1.00	-0.09 (-0.37, 0.19)	-0.05 (-0.21, 0.10)	0.92

GDM, gestational diabetes mellitus.

BMI=Body Mass Index; TST=Triceps skinfold thickness z-score; SST=Subscapular skinfold thickness; SSFT=sum of skinfold thicknesses; HDL=High density lipoprotein; LDL=Low density lipoprotein; SGA = small for gestational age; AGA = appropriate for gestational age; LGA = large for gestational age.

Model 1 adjusted for maternal age, parity, educational level, pre-pregnancy BMI, GDM, pregnancy-induced hypertension, child sex; Model 2 adjusted for maternal age, parity, educational level, pre-pregnancy BMI, GDM, pregnancy-induced hypertension, child sex, birth weight.

Table S2. Stratified associations between cord blood metabolic factors and childhood adiposity indicators by BMI z-score

Cord blood lipids	Regression coefficients (95% confidence interval)			P interaction		
	Underweight (n=312)	Normal weight (n=861)	Overweight/obese (n=145)	Underweight vs Normal weight	Overweight vs Normal weight	Underweight vs Overweight/obese
**Total cholesterol**						
Weight-for-length z-score	0.10 (-0.03, 0.23)	0.01 (-0.05, 0.08)	0.06 (-0.12, 0.24)	0.22	0.47	0.83
BMI-for-age z-score	0.11 (-0.03, 0.24)	0.01 (-0.06, 0.08)	0.05 (-0.13, 0.23)	0.19	0.50	0.76
TST-for-age z-score	0.04 (-0.13, 0.21)	0.03 (-0.07, 0.13)	0.33 (-0.03, 0.68)	0.83	**0.02**	**0.03**
SST-for-age z-score	0.19 (-0.05, 0.43)	0.01 (-0.12, 0.13)	0.36 (-0.18, 0.90)	0.21	0.09	0.46
SSFT-for-age z-score	0.08 (-0.11, 0.26)	0.07 (-0.12, 0.27)	0.43 (-0.05, 0.92)	0.77	0.25	0.23
**Triglycerides**						
Weight-for-length z-score	-0.02 (-0.15, 0.10)	-0.07 (-0.13, 0.00)	-0.23 (-0.41, -0.04)	0.54	0.24	0.15
BMI-for-age z-score	-0.01 (-0.14, 0.11)	-0.07 (-0.13, 0.00)	-0.22 (-0.41, -0.03)	0.45	0.25	0.13
TST-for-age z-score	-0.11 (-0.29, 0.06)	-0.03 (-0.12, 0.07)	0.00 (-0.36, 0.36)	0.24	0.73	0.66
SST-for-age z-score	-0.21 (-0.45, 0.04)	-0.06 (-0.18, 0.06)	0.11 (-0.41, 0.63)	0.16	0.23	0.05
SSFT-for-age z-score	-0.17 (-0.34, 0.02)	-0.15 (-0.32, 0.03)	0.04 (-0.43, 0.52)	0.80	0.40	0.38
**HDL**						
Weight-for-length z-score	0.06 (-0.06, 0.19)	0.06 (-0.01, 0.13)	0.07 (-0.14, 0.28)	0.97	0.97	1.00
BMI-for-age z-score	0.06 (-0.07, 0.18)	0.06 (-0.01, 0.12)	0.06 (-0.15, 0.28)	1.00	0.96	0.97
TST-for-age z-score	0.02 (-0.14, 0.17)	0.09 (-0.01 ,0.19)	0.31 (-0.04, 0.67)	0.37	**0.02**	**<0.01**
SST-for-age z-score	0.09 (-0.13, 0.32)	0.05 (-0.08, 0.17)	0.24 (-0.30, 0.78)	0.63	0.43	0.68
SSFT-for-age z-score	0.02 (-0.15, 0.19)	0.17 (-0.03, 0.36)	0.31 (-0.17, 0.79)	0.29	0.61	0.27
**LDL**						

Weight-for-length z-score	0.13 (0.00, 0.27)	0.00 (-0.07, 0.06)	0.06 (-0.10, 0.22)	0.07	0.28	0.69
BMI-for-age z-score	0.14 (0.00, 0.27)	-0.01 (-0.07, 0.06)	0.06 (-0.10, 0.22)	**0.057**	0.29	0.61
TST-for-age z-score	0.01 (-0.17, 0.18)	-0.02 (-0.11, 0.08)	0.20 (-0.14, 0.54)	0.98	0.22	0.27
SST-for-age z-score	0.19 (-0.05, 0.43)	0.02 (-0.11, 0.14)	0.24 (-0.30, 0.78)	0.27	0.15	0.58
SSFT-for-age z-score	0.07 (-0.12, 0.25)	-0.09 (-0.28, 0.11)	0.36 (-0.10, 0.82)	0.55	0.17	0.40

BMI=Body Mass Index; TST=Triceps skinfold thickness z-score; SST=Subscapular skinfold thickness; SSFT=sum of skinfold thicknesses; HDL=High density lipoprotein; LDL=Low density lipoprotein.

Adjusted for maternal age, parity, educational level, pre-pregnancy BMI, GDM, pregnancy-induced hypertension, child sex, birth weight.

Table S3. Stratified associations between cord blood metabolic factors and childhood adiposity indicators by BMI z-score, additionally adjusted for birth weight z-score

Cord blood lipids	Regression coefficients (95% confidence interval)			P interaction		
	Underweight (n=312)	Normal weight (n=861)	Overweight/obese (n=145)	Underweight vs Normal weight	Overweight vs Normal weight	Underweight vs Overweight/obese
**Total cholesterol**						
Weight-for-length z-score	0.08 (-0.05, 0.20)	0.00 (-0.06, 0.06)	0.03 (-0.14, 0.21)	0.28	0.57	0.81
BMI-for-age z-score	0.08 (-0.05, 0.21)	0.00 (-0.07, 0.06)	0.03 (-0.15, 0.21)	0.24	0.59	0.75
TST-for-age z-score	0.04 (-0.14, 0.21)	0.03 (-0.07, 0.13)	0.34 (-0.03, 0.70)	0.83	**0.02**	**0.03**
SST-for-age z-score	0.17 (-0.07, 0.41)	0.00 (-0.13, 0.12)	0.34 (-0.23, 0.91)	0.20	0.10	0.52
SSFT-for-age z-score	0.06 (-0.12, 0.25)	0.06 (-0.13, 0.26)	0.44 (-0.08, 0.95)	0.79	0.25	0.26
**Triglycerides**						
Weight-for-length z-score	0.09 (-0.04, 0.22)	0.01 (-0.06, 0.08)	-0.19 (-0.38, -0.01)	0.40	0.13	0.06
BMI-for-age z-score	0.09 (-0.04, 0.22)	0.01 (-0.06, 0.07)	-0.19 (-0.39, 0.00)	0.34	0.15	0.06
TST-for-age z-score	-0.10 (-0.29, 0.08)	-0.01 (-0.11, 0.08)	0.00 (-0.38, 0.37)	0.24	0.72	0.68
SST-for-age z-score	-0.15 (-0.40, 0.10)	-0.02 (-0.14, 0.10)	0.15 (-0.39, 0.68)	0.16	0.25	0.06
SSFT-for-age z-score	-0.13 (-0.32, 0.07)	-0.12 (-0.31, 0.07)	0.07 (-0.42, 0.55)	0.80	0.40	0.39
**HDL**						
Weight-for-length z-score	0.02 (-0.11, 0.14)	0.03 (-0.03, 0.10)	0.03 (-0.18, 0.24)	0.79	0.94	0.93
BMI-for-age z-score	0.02 (-0.11, 0.14)	0.03 (-0.03, 0.10)	0.03 (-0.18, 0.25)	0.79	0.39	0.91
TST-for-age z-score	0.01 (-0.15, 0.17)	0.08 (-0.02, 0.18)	0.31 (-0.05, 0.68)	0.37	**0.02**	**<0.01**
SST-for-age z-score	0.07 (-0.15, 0.30)	0.03 (-0.10, 0.16)	0.22 (-0.33, 0.77)	0.60	0.43	0.70

SSFT-for-age z-score	0.01 (-0.17, 0.18)	0.15 (-0.04, 0.34)	0.30 (-0.19, 0.79)	0.31	0.61	0.27
**LDL**						
Weight-for-length z-score	0.11 (-0.02, 0.25)	-0.02 (-0.08, 0.05)	0.05 (-0.10, 0.21)	0.08	0.27	0.71
BMI-for-age z-score	0.12 (-0.01, 0.25)	-0.02 (-0.08, 0.05)	0.05 (-0.11, 0.21)	0.06	0.30	0.62
TST-for-age z-score	0.00 (-0.17, 0.18)	-0.02 (-0.12, 0.08)	0.21 (-0.14, 0.56)	0.97	0.23	0.28
SST-for-age z-score	0.17 (-0.07, 0.41)	0.01 (-0.12, 0.13)	0.21 (-0.36, 0.78)	0.27	0.19	0.64
SSFT-for-age z-score	0.05 (-0.14, 0.23)	-0.09 (-0.29, 0.10)	0.36 (-0.14, 0.85)	0.56	0.17	0.45

BMI=Body Mass Index; TST=Triceps skinfold thickness z-score; SST=Subscapular skinfold thickness; SSFT=sum of skinfold thicknesses; HDL=High density lipoprotein; LDL=Low density lipoprotein.

Adjusted for maternal age, parity, educational level, pre-pregnancy BMI, GDM, pregnancy-induced hypertension, child sex, birth weight.

Table S4. Associations between cord blood metabolic factors and childhood adiposity indicators after excluding premature infants.

Cord blood lipids	Model 1		Model 2	
	Beta (95% CI)	P	Beta (95% CI)	P
**Total cholesterol**				
Weight-for-length z-score	0.04 (-0.01, 0.10)	0.14	0.03 (-0.03, 0.08)	0.39
BMI-for-age z-score	0.04 (-0.02, 0.10)	0.16	0.03 (-0.03, 0.08)	0.39
TST-for-age z-score	0.07 (-0.02, 0.16)	0.11	0.07 (-0.02, 0.15)	0.14
SST-for-age z-score	0.08 (-0.03, 0.19)	0.16	0.07 (-0.04, 0.18)	0.24
SSFT-for-age z-score	0.12 (-0.03, 0.27)	0.11	0.11 (-0.04, 0.26)	0.15
**Triglycerides**				
Weight-for-length z-score	-0.08 (-0.14, -0.03)	**0.01**	0.00 (-0.06, 0.06)	0.94
BMI-for-age z-score	-0.08 (-0.13, -0.02)	**0.01**	-0.01 (-0.07, 0.05)	0.84
TST-for-age z-score	-0.05 (-0.13, 0.03)	0.25	-0.04 (-0.12, 0.05)	0.40
SST-for-age z-score	-0.09 (-0.19, 0.02)	0.10	-0.05 (-0.16, 0.06)	0.34
SSFT-for-age z-score	-0.14 (-0.28, 0.00)	0.05	-0.11 (-0.26, 0.04)	0.14
**HDL**				
Weight-for-length z-score	0.07 (0.01, 0.13)	**0.02**	0.04 (-0.02, 0.10)	0.20
BMI-for-age z-score	0.07 (0.01, 0.12)	**0.03**	0.04 (-0.02, 0.10)	0.21
TST-for-age z-score	0.10 (0.02, 0.18)	**0.02**	0.09 (0.01, 0.18)	**0.03**
SST-for-age z-score	0.07 (-0.03, 0.18)	0.18	0.06 (-0.05, 0.17)	0.28
SSFT-for-age z-score	0.15 (0.01, 0.29)	0.04	0.14 (-0.01, 0.28)	0.06
**LDL**				
Weight-for-length z-score	0.04 (-0.02, 0.10)	0.22	0.03 (-0.03, 0.08)	0.39
BMI-for-age z-score	0.04 (-0.02, 0.10)	0.23	0.03 (-0.03, 0.08)	0.39
TST-for-age z-score	0.01 (-0.08, 0.10)	0.82	0.01 (-0.08, 0.09)	0.89
SST-for-age z-score	0.08 (-0.03, 0.19)	0.15	0.07 (-0.04, 0.18)	0.23
SSFT-for-age z-score	-0.01 (-0.16, 0.14)	0.92	-0.02 (-0.17, 0.13)	0.80

BMI=Body Mass Index; TST=Triceps skinfold thickness z-score; SST=Subscapular skinfold thickness; SSFT=sum of skinfold thicknesses; HDL=High density lipoprotein; LDL=Low density lipoprotein.

Model 1 adjusted for maternal age, parity, educational level, pre-pregnancy BMI, GDM, pregnancy-induced hypertension, child sex; Model 2 adjusted for maternal age, parity, educational level, pre-pregnancy BMI, GDM, pregnancy-induced hypertension, child sex, birth weight.

Table S5. Associations between cord blood metabolic factors and childhood adiposity indicators after excluding women with pregnancy-induced hypertension.

Cord blood lipids	Model 1		Model 2	
	Beta (95% CI)	P	Beta (95% CI)	P
**Total cholesterol**				
Weight-for-length z-score	0.04 (-0.01, 0.10)	0.14	0.02 (-0.04, 0.08)	0.47
BMI-for-age z-score	0.04 (-0.02, 0.10)	0.15	0.02 (-0.03, 0.08)	0.45
TST-for-age z-score	0.06 (-0.03, 0.14)	0.17	0.055 (-0.03, 0.14)	0.20
SST-for-age z-score	0.08 (-0.03, 0.19)	0.17	0.06 (-0.05, 0.17)	0.25
SSFT-for-age z-score	0.13 (0.00, 0.27)	0.06	0.12 (-0.01, 0.26)	0.08
**Triglycerides**				
Weight-for-length z-score	-0.08 (-0.13, -0.02)	**0.01**	0.00 (-0.06, 0.06)	0.96
BMI-for-age z-score	-0.07 (-0.13, -0.01)	**0.02**	0.00 (-0.06, 0.06)	0.99
TST-for-age z-score	-0.06 (-0.14, 0.02)	0.17	-0.05 (-0.13, 0.04)	0.26
SST-for-age z-score	-0.09 (-0.19, 0.02)	0.10	-0.05 (-0.16, 0.06)	0.36
SSFT-for-age z-score	-0.15 (-0.28, -0.02)	**0.03**	-0.12 (-0.26, 0.02)	0.09
**HDL**				
Weight-for-length z-score	0.07 (0.02, 0.13)	**0.01**	0.04 (-0.02, 0.10)	0.16
BMI-for-age z-score	0.07 (0.01, 0.13)	**0.02**	0.04 (-0.02, 0.10)	0.16
TST-for-age z-score	0.09 (0.01, 0.17)	**0.03**	0.09 (0.01, 0.17)	**0.04**
SST-for-age z-score	0.07 (-0.03, 0.18)	0.18	0.06 (-0.05, 0.16)	0.29
SSFT-for-age z-score	0.16 (0.03, 0.30)	**0.02**	0.15 (0.02, 0.28)	**0.03**
**LDL**				
Weight-for-length z-score	0.03 (-0.03, 0.09)	0.32	0.01 (-0.04, 0.07)	0.64
BMI-for-age z-score	0.03 (-0.03, 0.09)	0.33	0.01 (-0.042, 0.07)	0.63
TST-for-age z-score	0.01 (-0.08, 0.09)	0.90	0.00 (-0.08, 0.09)	0.96
SST-for-age z-score	0.08 (-0.039, 0.19)	0.17	0.06 (-0.05, 0.17)	0.25
SSFT-for-age z-score	0.00 (-0.14, 0.14)	0.98	-0.01 (-0.15, 0.13)	0.90

BMI=Body Mass Index; TST=Triceps skinfold thickness z-score; SST=Subscapular skinfold thickness; SSFT=sum of skinfold thicknesses; HDL=High density lipoprotein; LDL=Low density lipoprotein.

Model 1 adjusted for maternal age, parity, educational level, pre-pregnancy BMI, GDM, pregnancy-induced hypertension, child sex; Model 2 adjusted for maternal age, parity, educational level, pre-pregnancy BMI, GDM, pregnancy-induced hypertension, child sex, birth weight.

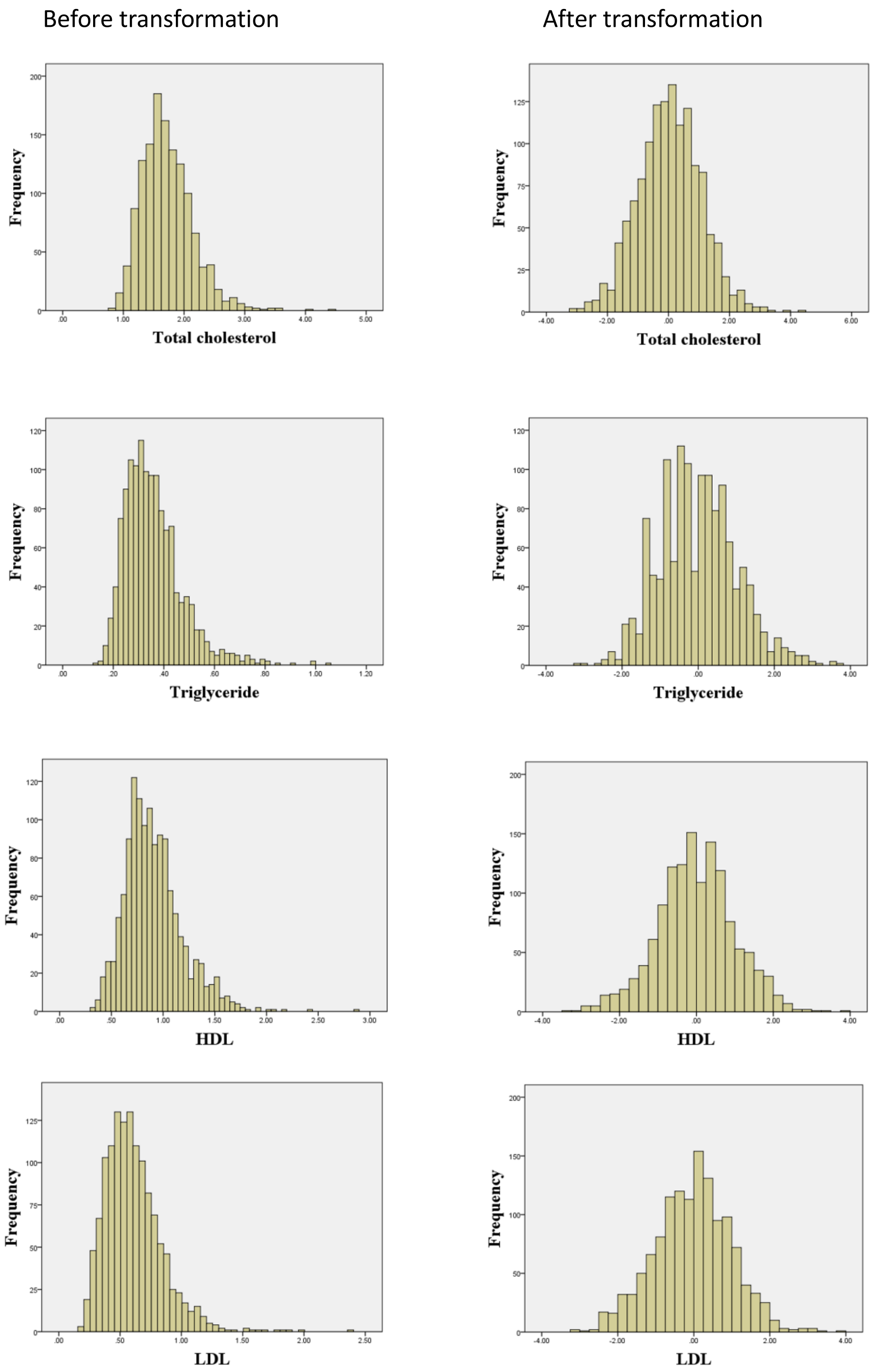

Figure S1. Distribution of lipid measures before and after transformation
